# The Impact of the COVID-19 Pandemic on Melanoma Diagnosis: A Single-Center Study

**DOI:** 10.3390/diagnostics14182032

**Published:** 2024-09-13

**Authors:** Adrian-Horațiu Sabău, Iuliu-Gabriel Cocuz, Raluca Niculescu, Andreea Cătălina Tinca, Andreea Raluca Szoke, Bianca-Andreea Lazar, Diana Maria Chiorean, Ovidiu Simion Cotoi

**Affiliations:** 1 Doctoral School of Medicine and Pharmacy, University of Medicine, Pharmacy, Sciences and Technology “George Emil Palade” of Targu Mures, 540142 Targu Mures, Romania; adrian-horatiu.sabau@umfst.ro; 2 Pathology Department, Mures Clinical County Hospital, 540011 Targu Mures, Romania; raluca.niculescu@umfst.ro (R.N.); andreea.tinca@umfst.ro (A.C.T.); andreea.szoke@umfst.ro (A.R.S.); ohii.bianca@yahoo.com (B.-A.L.); diana.chiorean@umfst.ro (D.M.C.); ovidiu.cotoi@umfst.ro (O.S.C.); 3 Pathophysiology Department, University of Medicine, Pharmacy, Sciences and Technology “George Emil Palade” of Targu Mures, 540142 Targu Mures, Romania

**Keywords:** melanoma, COVID-19, histopathology, epidemiology

## Abstract

(1) Background: Melanoma represents the most aggressive form of skin cancer, with an increasing incidence and numerous risk factors. The COVID-19 pandemic has led to modifications in work protocols. This study aims to elucidate potential changes in the number and characteristics of primary melanomas diagnosed in the Pathology Department of the Târgu Mureș County Clinical Hospital before, during, and after the COVID-19 pandemic. (2) Methods: This study included 140 patients grouped into six periods: two pre-COVID-19 periods, the COVID-19 pandemic period, and three post-COVID-19 periods. Epidemiological data, diagnoses, and histopathological reports were analyzed. (3) Results: The number of cases diagnosed during the COVID-19 pandemic was significantly lower than in the other analyzed periods. Regarding the monitored parameters, a statistically significant increase was observed in the first two post-COVID-19 periods, with a return to values similar to those of the first period in the last analyzed period. Additionally, a statistically significant increase in the incidence of distant metastases was identified in the post-COVID-19 periods. (4) Conclusions: The COVID-19 pandemic had a negative impact on the diagnosis of new melanoma cases, leading to an increase in the number of cases and a worsening of prognostic parameters.

## 1. Introduction

Melanoma represents the most aggressive form of skin cancer, drawing the attention of specialists and public health institutes worldwide. According to the World Health Organization (WHO), the incidence of melanoma has significantly increased in recent decades, particularly in developed countries, making its prevention, diagnosis, and treatment global priorities [1,2]. Risk factors, such as excessive sun exposure or the improper use of tanning beds, have been identified and highlighted by the WHO, emphasizing the importance of adequate awareness and education [3,4,5].

According to the American Cancer Society, the incidence of melanoma in the past 10 years has shown an annual increase of 2–3%, with a projected 100,640 new cases of melanoma in situ and 8290 melanoma-related deaths in the United States in the year 2024 [6]. In Europe, 150,000 new cases of melanoma are reported annually, with the distribution and timing of diagnosis being uneven due to delays in diagnosis, underdeveloped screening programs, and funding shortages in Southern and Eastern European countries [7].

According to the eighth AJCC classification of melanoma, approximately 90% of newly diagnosed cases do not present with metastases at the time of diagnosis [8]. The most important histopathological factors are the vertical thickness of the tumor (Breslow index), the presence or absence of ulceration, the number of mitoses per 10 HPF, and the level of invasion (Clark level) [9].

SARS-CoV-2, the virus responsible for the COVID-19 pandemic, has resulted in particularly high morbidity and mortality rates for elderly patients and those with comorbidities such as obesity; diabetes; cardiovascular diseases, including cardiomyopathy; and cancer [10,11]. Multiple studies have evaluated the impact of COVID-19 on patients with various types of cancer and those already undergoing oncological treatment [1,12].

In March 2020, the World Health Organization declared Coronavirus 2019 (COVID-19) a global pandemic. Subsequently, a wide range of measures were implemented worldwide to limit the spread of the virus. Several governments and national authorities imposed so-called lockdowns and quarantines, during which the population was advised to avoid leaving their homes and to postpone non-urgent medical visits and emergency services use.

Beyond the serious economic and social consequences, the negative impact of these regulations on the standards of medical care became evident in various sectors of national health systems and in different medical disciplines, including those not directly involved in managing COVID-19 patients, which were affected collaterally [13].

The present study extends the research previously conducted at our center towards the end of the COVID-19 pandemic, which highlighted a notable decrease in the number of newly diagnosed cases of cutaneous malignant lesions. Specifically, this earlier research identified a reduction in both non-melanocytic skin cancers and melanocytic skin cancers during that period [14]. In this context, our study was designed to investigate and clarify potential changes in both the quantity and characteristics of primary melanomas, as well as the incidence of metastases and local recurrences, diagnosed within our Pathology Department. We sought to analyze these variables across three distinct time periods: before, during, and after the COVID-19 pandemic. By examining the data from these different phases, our study aimed to identify any shifts or trends in the incidence of primary melanoma cases, assess how the pandemic might have influenced the presentation or progression of these cases, and evaluate the rates of metastases and local recurrences during each period. This comprehensive analysis provides insights into how the pandemic impacted the diagnosis and progression of melanoma, offering a clearer picture of its effects on patient outcomes and informing future clinical and diagnostic strategies.

## 2. Materials and Methods

A cross-sectional study was carried out by analyzing diagnostic data from the Clinical Pathology Department of the Târgu Mureș County Clinical Hospital in Romania. This analysis covered a period extending from April 2018 to January 2024.

Surgical and biopsy excisions were sourced from the Departments of Surgery, Plastic and Reconstructive Surgery and Dermatology at the Târgu Mureș County Clinical Hospital. Diagnostic data were collected from the department’s electronic database and checked by two pathologists for accuracy. In our database, we have included patients’ epidemiological data (age, sex, and origin), histopathological diagnoses, and histopathological reports, which included the Breslow index, tumor ulceration, Clark level of invasion, tumor pTNM stage, number of intratumoral mitoses per 10 HPF, the Ki-67 proliferation index, and the presence of invasions (lympho-vascular and perineural). Local recurrences and distant metastases were also included. The exclusion criteria comprised cases referred from clinical services outside of the Târgu Mureș County Clinical Hospital, the absence of a parameter in the histopathological report, and the lack of a prior diagnosis for local recurrences and distant metastases.

To avoid and limit possible inter-observer errors, all diagnosed cases were evaluated according to the standardized working protocols of the Department of Pathology and were handled by the same team of pathologists.

Six study periods were defined: April 2018–March 2019 (Period 1 (P1)—before the COVID-19 pandemic), April 2019–February 2020 (Period 2 (P2)—before the COVID-19 pandemic), March 2020–January 2021 (Period 3 (P3)—during the COVID-19 pandemic), February 2021–January 2022 (Period 4 (P4)—after the COVID-19 pandemic), February 2022–January 2023 (Period 5 (P5)—after the COVID-19 pandemic), and February 2023–January 2024 (Period 6 (P6)—after the COVID-19 pandemic).

For the statistical analysis, MS EXCEL 2023, GraphPad Prism 8, and IBM SPSS version 27.0 software were utilized. Grubbs’ test was used to identify and eliminate outliers. The Kolmogorov–Smirnov test was applied to determine data normality before applying parametric (Pearson) and non-parametric (Wilcoxon) tests.

This study was conducted in accordance with the Declaration of Helsinki and was approved by the hospital’s ethics committee (protocol number 2200/15.03.2021).

## 3. Results

During the study period, according to the inclusion criteria, a total of 140 patients (77 men and 63 women) were included. Eighteen cases were excluded based on the exclusion criteria. A total of 81 patients were registered with primary cutaneous melanoma, and 59 with metastases and recurrences. No statistically significant difference was observed regarding the mean age at diagnosis, which was 66 years old for the patients from the pre-COVID-19 periods (Periods 1 and 2), 68 years old for the patients during the COVID-19 period (Period 3), and 64 years old for the patients from the post-COVID-19 periods (Periods 4, 5, and 6). Additionally, no statistically significant changes were observed concerning the patients’ sex and origin.

In the first two analyzed periods (Periods 1 and 2, pre-COVID-19 pandemic periods), 27 cases of primary cutaneous melanoma were identified, whereas during the COVID-19 period (Period 3), only 4 new cases were identified. A statistically significant increase in the average number of atypical mitoses (*p* < 0.005) and a statistically significant increase in the Ki-67 proliferation index (*p* < 0.005) were observed (Table 1).

In the first two post-COVID-19 periods, 32 newly diagnosed cases of cutaneous melanoma were identified. Statistically significant increases were observed in the average Breslow index (*p* < 0.005), the number of atypical mitoses (*p* < 0.005), and the Ki-67 proliferation index (*p* < 0.005) compared to the pre-COVID-19 periods and the levels seen during the COVID-19 period (Table 1 and Table 2).

In the sixth analyzed period (February 2023–January 2024), two years after the COVID-19 pandemic, 18 new cases of cutaneous melanoma were identified. A statistically significant decrease (*p* < 0.005) in the average Breslow index was observed compared to the pre-COVID-19 periods and the levels seen during the COVID-19 period (Table 3).

Additionally, we observed a decrease in the Clark level during the COVID-19 period compared to the pre-COVID-19 periods (P1 + P2: 3.37; P3: 3). In the first two post-COVID periods, an increase in the Clark level was noted, followed by a decrease to a value close to that of the pre-COVID-19 periods (P4 + P5: 3.71; P6: 3.33). There were increases in the presence of ulceration, lympho-vascular invasion, and neural invasion in the post-COVID-19 periods.

Regarding the metastases identified in the analyzed periods, there was a significant increase in the number of cases in the post-COVID-19 periods. Out of the total 44 cases, the majority were identified in the axillary (n = 24) and inguinal (n = 10) lymph nodes. The remaining cases consisted of secondary determinations in the lungs, pleura, and gastrointestinal and hepatic regions. The incidence of local recurrences did not present significant changes across the analyzed periods (Table 4).

## 4. Discussion

The COVID-19 pandemic necessitated a rapid adaptation in medical services, requiring modified work protocols and hospital circuits, and separate wards for patients diagnosed with SARS-CoV-2 and those without infection [15]. Due to these changes, the hospitalization of oncology patients decreased, and undiagnosed patients experienced delays in diagnosis and treatment administration [16]. The diagnosis of patients with melanoma typically begins with a straightforward skin examination, a crucial step in early detection. However, for many patients, this examination was delayed because of protective measures implemented during the COVID-19 pandemic. These protective protocols, designed to limit exposure and spread of the virus, inadvertently led to postponements in routine medical visits, including essential skin checks. Consequently, the timely identification and diagnosis of melanoma were significantly impacted, potentially delaying treatment and affecting patient outcomes [17].

Studies conducted towards the end of the COVID-19 pandemic observed a notable decrease in the number of diagnosed cases across various medical conditions. These findings led to the anticipation of a potential surge in new cases in the periods following the pandemic. The expectation is that the backlog of undiagnosed or untreated conditions, combined with the resumed access to healthcare services, might result in an increase in diagnoses as healthcare systems catch up [18,19].

Numerous recent studies analyzing the epidemiological and histopathological data of patients diagnosed with melanoma, including meta-analyses and individual studies from Europe and various centers in Romania, highlighted an increase in newly diagnosed cases and worsened monitored parameters (the Breslow index, the presence of ulceration, Clark Level, and number of atypical mitoses) [20,21,22,23]. The results we have obtained align closely with the findings from other studies. This suggests that the results observed in our research are not only valid but are also corroborated by similar outcomes in other investigations. The consistency between these findings and ours reinforces the reliability of our study’s results and contributes to the broader understanding of the subject matter. This alignment further implies that our research may be on the right track, as it mirrors what has been observed by others in the field. Another possible cause for the observed changes, apart from the delayed diagnosis due to protective measures, could be the psychological stress experienced by patients and the fear of a possible infection within the hospital environment.

A particularly important aspect of our study is the analysis of the sixth period (February 2023–January 2024). The data from this period reveal a reduction in the analyzed parameters when compared to the previous two post-COVID-19 periods. This decrease reaches values similar to the pre-COVID-19 periods without statistically significant differences. Through this analysis and the dynamic changes in the monitored parameters, we once again highlight the negative impact of the COVID-19 pandemic on newly diagnosed patients with melanoma and on patients who experienced delayed therapy or inadequate follow-up. Two possible causes for this normalization could be the relaxation of protective measures, which led to the resumption of routine medical activities, and the increase in vaccination rates among the population, which resulted in a decrease in the number of cases and the normalization of access to healthcare services.

Due to the protective measures adopted during the COVID-19 pandemic and the new work protocols that were introduced, patient follow-up decreased, which led to an increase in the incidence of metastases and recurrences in the periods following the pandemic. This is clearly demonstrated in our study, which reveals a markedly higher occurrence of metastases detected during the post-COVID-19 periods. The data indicate that the rate at which metastases were identified increased significantly after the onset of the COVID-19 pandemic.

The COVID-19 pandemic posed an unparalleled challenge for hospitals, as well as for clinical and surgical departments, including Pathology Services. The pandemic’s unprecedented nature meant that healthcare systems had to navigate an overwhelming surge in the number of patients, while simultaneously adapting to new protocols designed to curb the virus’s spread. This situation had a profound impact on the delivery of medical services, necessitating swift and substantial changes to existing practices.

Hospitals faced logistical hurdles such as reallocating resources, managing staff shortages, and implementing infection control measures. Clinical and surgical departments had to adjust their operations to prioritize emergency and COVID-19-related cases, which often led to delays or alterations in non-COVID-related care. Pathology Services, responsible for diagnosing diseases through laboratory tests, experienced disruptions in workflow, delays in processing samples, and challenges in maintaining accurate diagnoses amidst the crisis.

These rapid adaptations were crucial for managing the immediate health crisis, but they also highlighted the strain on healthcare systems, and the need for flexibility and resilience in the face of evolving situations. The pandemic underscored the importance of robust contingency plans and the ability to pivot quickly in response to unprecedented challenges.

In our region and at our medical center, the implemented measures aimed to limit the spread of infection. In this regard, clinical departments had a reduced number of patients, prioritizing acute cases, while surgical departments significantly decreased the number of surgical interventions. Additionally, numerous clinical departments (as was the case with the Dermatology department) were transformed into support services for the departments treating COVID-19 patients. Moreover, there was a massive reallocation of medical staff from non-COVID-19 departments to COVID-19 departments.

After the end of the COVID-19 pandemic, to address the delays in diagnosing and treating oncology patients, clinical services returned to normal workflows, surgical services prioritized interventions for oncology patients, and screening programs and dermatological consultations were intensified to diagnose delayed patients.

The lack of national cancer registries in many countries around the world still represents a significant medical issue, particularly when it comes to long-term patient follow-up, the maintenance of records of therapies, and systematic patient evaluation. In our country, the cancer registry was established in 2016, but its activity remains limited due to insufficient resources and underreporting in some regions. Additionally, a national cancer registry would greatly aid in drawing conclusions and results regarding the impact of the COVID-19 pandemic on oncology patients.

To be better prepared for similar situations in the future, it is essential to create clear working guidelines and protocols that standardize procedures and workflows in such scenarios. Additionally, there is a need for better management of resources, both material and financial, available to each hospital. Another element that could be extremely important in future situations is telemedicine. This could be applied to both patient diagnosis, through tele-dermatology and tele-pathology, and for surgical interventions that could be performed remotely, thereby minimizing human contact and limiting the spread of an infection.

The primary limitations of this study include the absence of comprehensive patient follow-up, which prevents a thorough analysis of long-term survival rates and the effectiveness of delayed treatment. Without the ongoing monitoring of patients, it is challenging to draw definitive conclusions about how these factors may influence outcomes over time. Regarding treatment, a comprehensive follow-up could offer valuable information about the differences in treatment efficacy between patients who received timely treatment and those who experienced a delay in diagnosis. Additionally, the study’s scope was restricted by the relatively small number of cases, which is a direct consequence of the research being conducted within a single medical center. Considering that the measures implemented to limit the spread of infection were the same throughout the entire healthcare system in Romania, the results, even if obtained from a small sample, could be generalized to the entire country.

## 5. Conclusions

A significant decrease in the number of diagnosed melanoma cases was observed during the COVID-19 pandemic compared to the preceding periods analyzed. This decrease had repercussions on patients with suspected melanoma, whose surgical interventions were delayed. Numerous parameters with negative prognostic implications in histopathological reports showed significant increases in the early post-COVID-19 periods (P4 + P5): the Breslow index, pTNM stage, the presence of lympho-vascular and perineural invasions, and the Ki-67 proliferation index were the most critical parameters that underwent negative changes. In the late post-COVID-19 period, the data indicate a return to values that closely resemble those observed before the COVID-19 pandemic.

The COVID-19 pandemic also represented an unprecedented challenge for hospitals and clinical and surgical departments, as well as for Pathology Services. It had a significant impact on how medical services are provided and required rapid adaptations to cope with the continuously evolving situation.

## Figures and Tables

**Table 1:** The characteristics of the cases included in the pre-COVID-19 periods and during the COVID-19 period.

Characteristics	Pre-COVID-19 P1 + P2 (n = 27)	During COVID-19 P3 (n = 4)
Average Breslow Index (mm)	4.83	4.43
Median Breslow Index (mm)	3.5 (0.5–17)	4.45 (0.8–19)
Ulceration—NO	13 (48.15%)	2 (50%)
Ulceration—YES	14 (51.85%)	2 (50%)
Average Mitotic Count	11.96 (0–36)	15.75 (1–26)
Average Ki-67 Index	14.03	30
Clark Level		
Average Clark Level	3.37	3
I	2 (7.41%)	0 (0%)
II	5 (18.52%)	1 (25%)
III	5 (18.52%)	2 (50%)
IV	11 (40.74%)	1 (25%)
V	4 (14.81%)	0 (0%)
pT Staging		
Tis	2 (7.41%)	0 (0%)
T1a	7 (25.93%)	1 (25%)
T1b	0 (0%)	0 (0%)
T2a	1 (3.7%)	0 (0%)
T2b	2 (741%)	1 (25%)
T3a	2 (7.41%)	0 (0%)
T3b	1 (3.7%)	0 (0%)
T4a	1 (3.7%)	1 (25%)
T4b	11 (40.74%)	1 (25%)
Vascular/Lymphatic Invasion—NO	26 (96.3%)	3 (75%)
Vascular/Lymphatic Invasion—YES	1 (3.7%)	1 (25%)
Neural invasion—NO	27 (100%)	3 (75%)
Neural invasion—YES	0 (0%)	1 (25%)

**Table 2:** The characteristics of the cases included during the COVID-19 period and in the post-COVID-19 periods.

Characteristics	During COVID-19 P3 (n = 4)	Post-COVID-19 P4 + p5 (n = 32)
Average Breslow Index (mm)	4.43	5.24
Median Breslow Index (mm)	4.45 (0.8–19)	3.4 (0.15–21)
Ulceration—NO	2 (50%)	18 (56.25%)
Ulceration—YES	2 (50%)	14 (43.75%)
Average Mitotic Count	15.75 (1–26)	21.15 (0–69)
Clark Level		
Average Clark Level	3	3.71
I	0 (0%)	1 (3.13%)
II	1 (25%)	6 (18.75%)
III	2 (50%)	1 (3.13%)
IV	1 (25%)	17 (53.13%)
V	0 (0%)	7 (21.88%)
pT Staging		
Tis	0 (0%)	0 (0%)
T1a	1 (25%)	4 (12.5%)
T1b	0 (0%)	1 (3.13%)
T2a	0 (0%)	2 (6.25%)
T2b	1 (25%)	1 (3.13%)
T3a	0 (0%)	5 (15.63%)
T3b	0 (0%)	6 (18.75%)
T4a	1 (25%)	7 (21.88%)
T4b	1 (25%)	6 (18.75%)
Vascular/Lymphatic Invasion—NO	3 (75%)	25 (78.13%)
Vascular/Lymphatic Invasion—YES	1 (25%)	7 (21.88%)
Neural invasion—NO	3 (75%)	29 (90.63%)
Neural invasion—YES	1 (25%)	3 (9.38%)
Average Ki-67 Index	30	41.82

**Table 3:** The characteristics of the cases included in the pre-COVID-19 periods and the last post-COVID-19 period.

Characteristics	Pre-COVID-19 P1 + P2 (n = 27)	Post-COVID-19 P6 (n = 18)
Average Breslow Index (mm)	4.83	2.98
Median Breslow Index (mm)	3.5 (0.5–17)	1.4 (0.15–11)
Ulceration—NO	13 (48.15%)	13 (72.22%)
Ulceration—YES	14 (51.85%)	5 (27.78%)
Average Mitotic Count	11.96 (0–36)	12.44 (3–27)
Clark Level		
Average Clark Level	3.37	3.33
I	2 (7.41%)	1 (5.56%)
II	5 (18.52%)	0 (0%)
III	5 (18.52%)	9 (50%)
IV	11 (40.74%)	8 (44.44%)
V	4 (14.81%)	0 (0%)
pT Staging		
Tis	2 (7.41%)	0 (0%)
T1a	7 (25.93%)	6 (33.33%)
T1b	0 (0%)	1 (5.56%)
T2a	1 (3.7%)	4 (22.22%)
T2b	2 (741%)	0 (0%)
T3a	2 (7.41%)	3 (16.67%)
T3b	1 (3.7%)	0 (0%)
T4a	1 (3.7%)	0 (0%)
T4b	11 (40.74%)	4 (22.22%)
Vascular/Lymphatic Invasion—NO	26 (96.3%)	16 (88.89%)
Vascular/Lymphatic Invasion—YES	1 (3.7%)	2 (11.11%)
Neural invasion—NO	27 (100%)	16 (88.89%)
Neural invasion—YES	0 (0%)	2 (11.12%)
Average Ki-67 Index	14.03	27.77

**Table 4:** The metastases and local recurrences identified during the study.

Period	Metastases	Local Recurrences
P1 (April 2018–March 2019)	3	4
P2 (April 2019–March 2020)	8	1
P3 (April 2020–February 2021)	3	2
P4 (March 2021–February 2022)	7	2
P5 (March 2022–February 2023)	9	3
P6 (March 2023–February 2024)	14	3

## Data Availability

All data produced here are available and can be produced upon request.
